# Bilateral carotid dissection due to Eagle syndrome: case report

**DOI:** 10.1590/1677-5449.202500082

**Published:** 2026-04-10

**Authors:** Anita dos Santos Cardoso, Yago Marcelino Maciel, Ruymara Candal Nogueira, Rafaela Tezza Matiola, Thais Pereira da Rosa, Fernando Topanotti Tarabay, Gregory Vinícius Périco, Brunella Flores Pupo

**Affiliations:** 1 Universidade do Extremo Sul Catarinense – UNESC, Criciúma, SC, Brasil.; 2 Faculdade Multivix, Cachoeiro de Itapemirim, ES, Brasil.; 3 Aura Neurologia, Criciúma, SC, Brasil.

**Keywords:** internal carotid artery dissection, stylo-carotid syndrome, craniofacial abnormalities, dissecção da artéria carótida interna, síndrome estiloide-carotídea, anormalidades craniofaciais

## Abstract

Eagle Syndrome (ES), characterized by elongation of the styloid process (ESP), is a rare condition with diverse symptoms. Among its complications, carotid dissection is a significant outcome requiring accurate diagnosis and intervention. This study describes a case of bilateral carotid dissection associated with ES, emphasizing diagnostic challenges, management, and therapeutic approaches. Conducted under Research Ethics Committee protocol 68193123.5.0000.0119 and approval number 6.028.970, it reports on a 38-year-old woman with a 5-day history of left-sided headache, otalgia, syncope, right-hand limitation, and visual blurring. MRI revealed left carotid bulb occlusion and recent ischemic events, while angio-CT confirmed bilateral carotid dissections. After clinical management failed, an intraoral surgical approach was chosen, resulting in full neurological recovery. CT confirmed ESP, and genetic testing was negative. This case highlights the need to consider ES in atypical cervicofacial symptoms, given its underdiagnosed incidence (~4% for ESP; ~0.16% for ES), reinforcing the importance of better clinical guidelines.

## Resumo

A Síndrome de Eagle (SE), caracterizada pelo alongamento do processo estiloide (APE), é uma condição rara com sintomas diversos. Entre suas complicações, dissecção carotídea é desfecho relevante que requer diagnóstico e intervenção precisos. Este estudo descreve caso de dissecção carotídea bilateral associada à SE, enfatizando desafios diagnósticos, manejo e abordagens terapêuticas. Realizado sob CAAE 68193123.5.0000.0119, aprovação 6.028.970, relata caso de mulher de 38 anos que apresentava cefaleia intensa à esquerda há 5 dias, otalgia, síncope, limitação da mão direita e turvação visual. Ressonância magnética revelou oclusão do bulbo carotídeo esquerdo e eventos isquêmicos recentes, enquanto angio-TC confirmou dissecções carotídeas bilaterais. Por falha no manejo clínico, optou-se pela abordagem cirúrgica intraoral, com recuperação neurológica completa. TC confirmou APE e teste genético foi negativo. Há necessidade de considerar a SE em sintomas cervicofaciais atípicos, dada a sua incidência subdiagnosticada (~4% para APE; ~0,16% para SE), reforçando a importância de melhores orientações clínicas.

## INTRODUCTION

Eagle syndrome (ES) consists of unilateral or bilateral elongation of the styloid processes with calcification of the stylohyoid ligaments.^1^ Originating from the lower part of the temporal bone, the styloid process takes on a slender and pointed characteristic, establishing crucial anatomical relationships, such as with the external and internal carotid arteries and surrounding muscles and ligaments.^2^

Incidence of ES ranges from 1.4 to 30%,^3^ but only about 4% of cases present with clinical symptoms. This suggests that the true prevalence of symptomatic ES is approximately 0.16%, affecting more women than men, with an age range of 20 to 40 years.^3,4^ Characteristically, it produces symptoms such as intermittent facial pain, dysphagia, otalgia, a sensation of a foreign body, or vagal neuralgia, among others.^5^ Symptoms arise or worsen with swallowing, chewing, tongue movements, head rotation, or palpation of the tonsillar fossa. Being nonspecific, they can be confused with other conditions, such as myofascial pain, pain of dental origin, and temporomandibular joint pain, among others.^3^

This pathology has two forms: “classic ES” caused by pressure on the glossopharyngeal nerve and other local structures, which usually appears after tonsillectomy, and “stylo-carotid syndrome,” caused by pressure on the carotid artery.^6^ Pressure on the carotid artery can cause dissection of the internal carotid artery (ICA) resulting from rupture of a layer of the arterial wall, leading to blood infiltration into the vessel wall, which can affect the subintimal and medial layers, causing stenosis or occlusion of the artery, or the subadventitial layer, resulting in aneurysm formation.^7^ The objective of this paper was to report a case of Eagle syndrome in a young female patient, emphasizing the diagnostic challenges and the importance of recognizing this condition due to its varied symptoms and potential vascular risks.

This article was conducted following the ethical guidelines established by the Research Ethics Committee under protocol 68193123.5.0000.0119 and approval number 6.028.970.

## CASE REPORT

A 38-year-old Caucasian woman sought care at an outpatient neurology service complaining of intense left-sided headache persisting for five days. Additionally, she reported otalgia, paresis in the right hand, and visual blurring, culminating in an episode of syncope.

The patient had a past medical history of thyroidectomy and had previously used combined oral contraceptives. No prior neurological conditions or connective tissue disorders were reported and no other comorbidities were identified. The thyroidectomy had been uneventful and she reported one previous low-risk pregnancy with a normal outcome.

On physical examination, the patient exhibited claudication of the right upper and lower limbs, without corresponding motor weakness or hemiparesis. Strength and tone were preserved in all extremities. None of the hallmark findings of Eagle syndrome, such as cervicofacial pain radiating to the ear, dysphagia, or tenderness along the styloid process, were observed.

**Figure 1:**
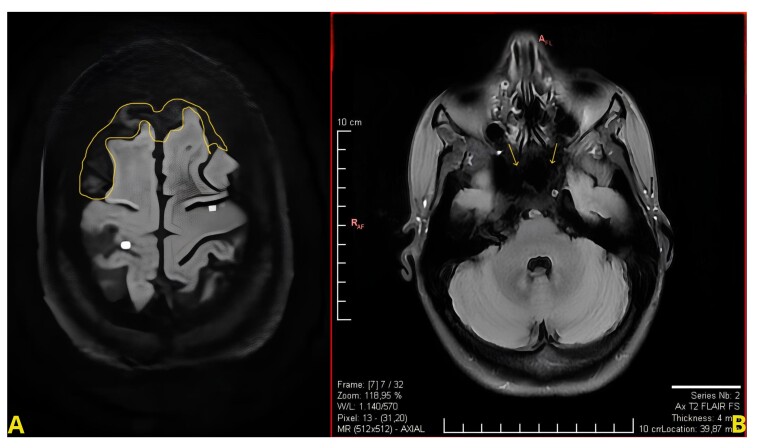
(A) Focal diffusion restriction in the frontal lobes bilaterally, suggestive of recent focal ischemic insults; (B) Absence of flow void in the extracranial internal carotid arteries, along with a lack of contrast enhancement in the left carotid bulb, indicating low flow or occlusion.

The initial assessment with magnetic resonance imaging revealed focal diffusion restriction in the frontal lobes bilaterally, indicating recent focal ischemic insults. Additionally, there was an absence of "flow-void" in the extracranial internal carotid arteries, coupled with a lack of contrast enhancement of the left carotid bulb, suggesting a possible low-flow state or occlusion (Figure 1).

**Figure 2:**
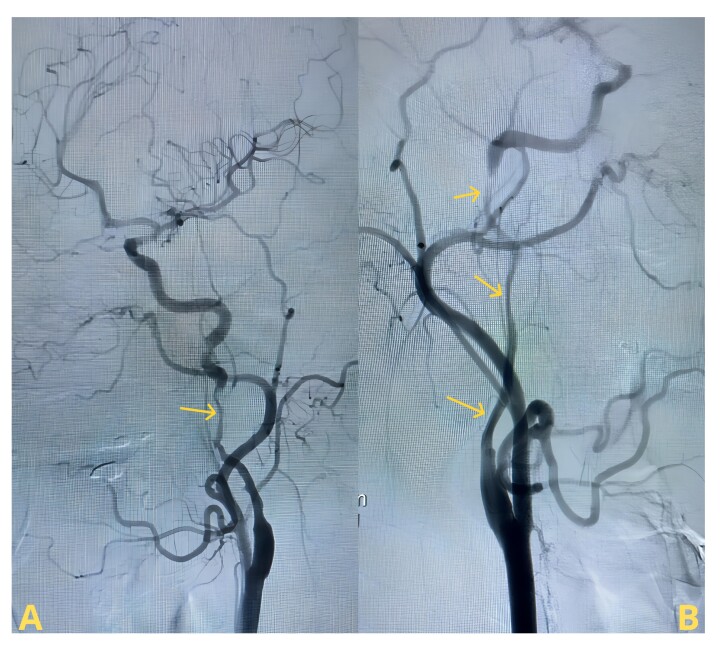
Cerebral arteriography image with internal carotid dissection. (A) Arrow indicates left carotid dissection; (B) Arrows indicate right carotid dissection. The left carotid bulb occlusion suggested in Figure 1A was not visible on arteriography, being only apparent on the previous angio-CT study.

Due to the suspicion of carotid dissection, the patient was referred to the emergency department, where dual antiplatelet therapy was immediately initiated. Subsequent arteriography (Figure 2) revealed severe dissections in both internal carotid arteries, with satisfactory arterial filling and the presence of vertebrobasilar collateral flow. No additional interventions were necessary. The patient experienced an excellent recovery and was discharged for further investigation of the etiology of the bilateral carotid dissection nine days after the procedure.

About two months after the first episode, the patient was readmitted due to a new episode of thunderclap headache. Venlafaxine hydrochloride was started for pain modulation, and a new arteriography was performed, presenting results similar to the first.

**Figure 3:**
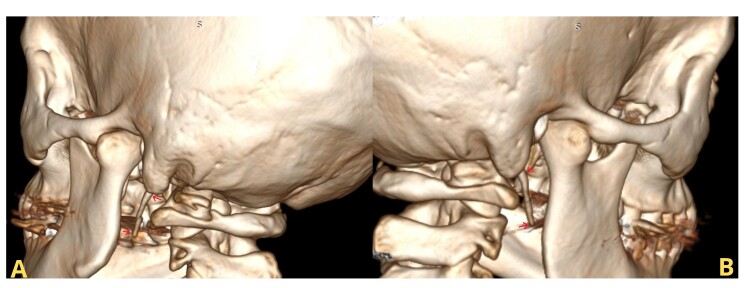
Three-dimensional reconstruction image from multislice computerized tomography scan. Posterolateral view of the elongated (A) left and (B) right styloid processes.

**Figure 4:**
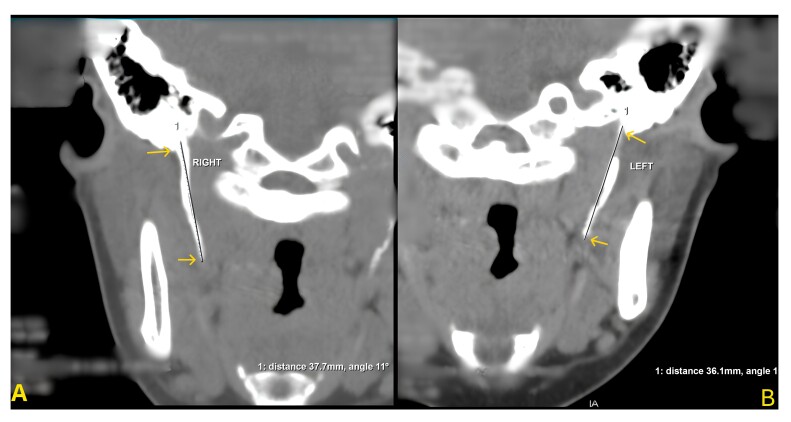
Two-dimensional images from multislice computerized tomography scan. Measures of elongated (A) right (37.7mm) and (B) left (36.1mm) styloid processes.

During outpatient follow-up, the genetic panel showed no alterations related to collagen diseases and the angio-CT revealed elongation of the styloid process on both the right (37.7 mm) and left (36.1 mm) sides (Figures 3-4), which very likely constitutes the underlying etiology of the patient’s carotid dissection, given the absence of other identifiable causes. The patient underwent elective intraoral resection of both elongated styloid processes under general anesthesia, performed by the oral and maxillofacial surgery team, for management of Eagle syndrome. The postoperative course was uneventful, and she was discharged without evident neurological sequelae. Before discharge, a follow-up angio-CT confirmed resolution of the condition. Postoperatively, the patient reported marked symptom improvement, with resolution of claudication, headache, otalgia, visual blurring, and paresthesia. There were no signs of recurrent dissection or neurological impairment at 6-month and 1-year follow-ups. Imaging follow-up was performed, and the control angio-CT at 1 year showed normal findings, confirming the stability of the dissection.

## DISCUSSION

Eagle Syndrome is a rare condition characterized by atypical ossification that affects a small portion of the population.^3^ The etiology of this syndrome is the subject of extensive debate in the literature, lacking clear consensus among specialists, making it an intriguing enigma for contemporary medicine.^2^

The styloid processes are slender bony projections originating from the temporal bone with a normal length of about 25 mm and are considered elongated when they exceed this measurement.^8^ As they extend inferomedially and become closely related to vascular and nervous structures, especially the internal carotid artery, the increased length can affect important adjacent structures, as demonstrated by the bilateral carotid dissection associated with ES described in this report.^9^

The clinical manifestations of ES present in a varied manner, manifesting as a wide array of chronic cervicofacial pain, often accompanied by symptoms such as dysphagia, odynophagia, ipsilateral otalgia, or retromandibular pain.^2^ Despite administration of various therapeutic techniques in attempts to mitigate the pain associated with this condition, clinical persistence is observed without effective resolution.

In the presence of an internal carotid artery dissection, ES should be considered as a possible cause, given that there is a specific treatment, styloidectomy, which has an estimated cure rate of 80%.^10^ In this regard, the “gold standard” method for diagnosing ES is computed tomography.^3^ Since this patient had a possible carotid artery dissection, angiotomography, which was conducted as part of her work-up, is the method of choice for visualizing the styloid process and enables evaluation of details of its angulation and anatomy.^10^

Currently, no definitive guidelines have been established for treatment of stylocarotid artery syndrome associated with a secondarily dissected carotid artery. Management strategies should be tailored to the specific clinical presentation, the severity of symptoms, and the presence of neurological deficits, since delaying treatment may result in deteriorating ischemic conditions or other serious complications.^11,12^

Recanalization interventions, including endovascular procedures, may be considered in line with treatment strategies for acute ischemic stroke.^12^ Furthermore, the presence of pseudoaneurysms or occlusive lesions secondary to the dissection may necessitate urgent surgical intervention or endovascular treatment.^13,14^

Conservative management includes analgesics, antidepressants, anticonvulsants, and corticosteroid or lidocaine injections via transpharyngeal administration. Additionally, non-steroidal anti-inflammatory drugs combined with local heat application may help alleviate symptoms associated with ES.^2,11^

In more complex cases, surgical intervention may be required, with evidence suggesting that styloidectomy is often necessary as a definitive treatment for symptomatic vascular Eagle syndrome, particularly in patients with recurrent cerebral infarctions due to carotid artery dissection.^2,11,15^ In the present case, surgical intervention was necessary, with the most effective approach being styloid process shortening. This can be performed through either an intraoral approach, the technique used in this case, or an extraoral approach, each having its own benefits and drawbacks, depending on the expertise of the treating professional.

## CONCLUSION

This case report describes a rare presentation of Eagle syndrome associated with bilateral internal carotid artery dissection. After unsuccessful conservative management, the patient underwent bilateral intraoral styloidectomy, resulting in complete neurological recovery and resolution of symptoms. Imaging follow-up demonstrated stability of the carotid dissections, with no evidence of recurrence. In this patient, recognition of ES as the underlying cause allowed definitive treatment and prevented further ischemic events.

## Data Availability

The data are not available due to legal and ethical restrictions. This case report involves identifiable clinical information, and public data sharing is restricted to ensure patient confidentiality and compliance with the approval granted by the Institutional Ethics Committee.
